# Four Cases of Resection of the Penis

**Published:** 1919

**Authors:** 


***Four Cases of Resection of the Penis.*** By J. de Sard. Note of an article to appear elsewhere.

In order to repair wounds of the genito-urinary organs, the operation should be performed in such a manner as to restore normal function as well as normal form. A period of preparation as well as one of action is recognized. The first includes the disinfection and cicatrization of wounds. In order to hasten this, it is especially important to divert the urinary stream in almost every case. Inasmuch as the urinary stream must be diverted for a long period, hypogastric drainage is much more certain and satisfactory than perineal drainage, and is, moreover, much more comfortable and heals more rapidly after withdrawing the tube.

The period of action includes one or several operations performed after complete cicatrization of the wounds. All scars are excised and the organ or organs are reconstructed if necessary by flaps adopted from the surrounding parts.

*Case I.* Wounded May 23rd, 1915. Transverse wound of penis 2 cms. from the peno-scrotal angle, with complete section of the urethra and of the right corpus cavernosum, and partial division of the left corpus cavernosum. The divided urethra is visible amid clots and gangrenous tissue on the stump. Urination is unimpeded. Several longitudinal incisions on the penis allow the sloughing tissues to be excised from the various wounds which are drained. Several shell fragments were removed and the wounds have healed. There results a complete occlusion of the distal cut end of the urethra, while the proximal end remains open.

Oct. 7. 1915. External urethrotomy for drainage. Dec. 17, 1915. Excision of perineal scar. The urethra is located by means of a sound. The excision requires the removal of about 0.5 cms. of tissue from the proximal stump. A sound is introduced into the proximal portion of the urethra and the mucous membrane freed by dissection. The two sounds are replaced by a single one introduced into the normal meatus and passed down into the bladder. Then the right corpus cavernosum is slit through, resected, and the sheaths of the corpora cavernosa united by several catgut sutures. Then the skin is united with silk. Convalescence uneventful. Primary union throughout. Jan. 3 1916. Perineal tube removed. Jan. 9. 1916. Urethra dilated to 27 F.

*Case II.* Wounded Jan. 21, 1916. Some form of an operation was performed and he arrived at the hospital on Jan. 30, with almost complete destruction of the lower part of the glans penis, considerable edema of the retracted prepuce, and a sutured wound 5 cms. long on the cut surface of the penis. The left testicle has been removed and this side of the scrotum shows a long sutured wound running up to the pubis in which there is a drain. Dorsal surface of the glans penis split into two unequal parts. July 1, 1916. Suprapubic cystotomy. After cicatrization penis shows grave deformity. Extensive loss of substance on the floor of the urethra. Aug. 18, 1916. The scar in the glans penis is cut away. The adjacent surfaces of the divided glans are removed from the urethra to the dorsum. The two portions of the glans penis are then readily approximated and sutured after tying two arteries. Sept. 20, 1916. The mucous membrane of the urethra was dissected free on a sound as a guide. The resection was carried backwards for about 2 cms. and the urethra thus freed was brought forward and fixed by several " O " silk sutures to the glans penis. The skin was then brought together beneath the urethra and finally sutured to the lower semi-circumference of the new meatus. Sept. 28, 1916. Sutures removed. Oct. 20, 1916. Suprapubic tube removed. Oct. 26, 1916. The shrunken glans penis shows a slight cicatrix with a urethral meatus which admits a large sized sound situated on its lower surface. Urination and erection entirely normal.

*Case IV.* Wounded Sept. 19, 1916. Entered hospital Nov. 29, 1916, after ligature of his right femoral artery, resection of the penis, and complete castration. On admission : deep wound on the right buttock and granulating wound of the suprapubic region with a small nubbin of skin on it which is the only vestige of the penis. Urination through the indwelling urethral catheter.

Nov. 30, 1916. Suprapubic cystotomy. Jan. 24, 1917. The wounds being cicatrized, a sound was introduced into the urethra and a horizontal incision about 8 cms. long carried across the pubic symphysis. Superficial tissues divided and suspensory ligament of the penis cut. The incision was then carried downward surrounding the urethral orifice and again downward to the base of the scrotum. The penis was then freed and depressed into the lower angle of the vertical scrotal incision. In order to reconstruct the sheath of the penis an overlying tab of skin is cut away. The scrotal skin is then sutured and the transverse incisions sutured vertically. A drain is left at the upper angle of the wound.

Feb. 27, 1917. In order to obtain a better stump and to facilitate urination, an ovoidal piece of skin is resected from the posterior surface of the scrotum. This piece is 5 cms. long and 3 cms. wide. The incision is sutured with "O" silk. The patient urinates readily and throws a good stream of urine. Urethra admits a 27 F. sound.

